# Physical Activity and Sedentary Behavior in Preterm-Born 7-Year Old Children

**DOI:** 10.1371/journal.pone.0155229

**Published:** 2016-05-11

**Authors:** John Lowe, W. John Watkins, Sarah J. Kotecha, Sailesh Kotecha

**Affiliations:** Department of Child Health, School of Medicine, Cardiff University, Cardiff, UK; Vanderbilt University, UNITED STATES

## Abstract

**Background:**

Previous studies of preterm-born children (<37 weeks’ gestation) have demonstrated decrements in lung function, exercise capacity, and increased respiratory symptoms compared to their term-born peers. However, it is unclear if these children have decreased levels of physical activity (PA) and increased sedentary behavior as a consequence of this increased respiratory morbidity. We therefore compared objectively measured PA in 7-year old preterm-born children with those born at term.

**Methods:**

Children in the Millennium Cohort Study underwent assessment of PA at 7 years of age using accelerometry. 6422/12781 (50%) provided valid accelerometry and had gestational age data. A series of general linear models adjusted for confounders investigated the association between gestational age and levels of Total PA (average accelerometer counts per minute over the period of the recording), Moderate-to-Vigorous PA (MVPA) and sedentary behavior. Mediation analysis was performed to specifically investigate whether the observed association of gestational age on PA was mediated by respiratory symptoms.

**Results:**

PA data were available for 79, 119, 275 and 5949 children born at 25–32, 33–34, 35–36 and 37–43 weeks’ gestation respectively. Boys born at ≤32 weeks’ gestation had modest but statistically significant reductions in MVPA when compared to term controls. This equated to a reduction of 9 minutes per day. No differences were found for Total PA or sedentary behavior. The association between gestational age and MVPA was not mediated by respiratory symptoms. In females, there was no association between gestational age and any measure of PA or sedentary behavior.

**Conclusions:**

Boys born at ≤32 weeks’ gestation took part in less MVPA than their term-born peers at 7 years of age. The differences were modest, but equated to a reduction of over 1 hour per week. Since PA levels have been shown to decline during childhood and adolescence, this vulnerable group deserves further surveillance.

## Introduction

Children born preterm (<37 weeks’ gestation) are at increased risk for respiratory-related morbidity [1, 2]. We have also shown reduced lung function of preterm-born school-age children using data from a large cohort study [3] as well as modest reductions in exercise capacity [4]. What is unclear is if such respiratory morbidities and decrements in lung function reduce children’s ability to participate in adequate levels of physical activity, possibly secondary to airway obstruction and impaired gas exchange. Physical activity (PA) is an important lifestyle choice which helps to ameliorate the risk of chronic disease however studies have shown that few children meet the recommended levels [5], with a steep decline occurring during adolescence. Preterm-born adults report reduced conditioning leisure time physical activity and reduced exercise capacity compared to those born at term [6–8]; since that respiratory morbidities track from childhood in to adulthood [9], investigating habitual physical activity participation in childhood may be of heightened importance for preterm-born children.

We recently reported on objectively measured levels of habitual PA in 11 and 15 year old children born preterm in the 1990s using data from the Avon Longitudinal Study of Parents and Children (ALSPAC) and did not find evidence of differences compared to children born at term [10], however, there were some methodological limitations including a possible underestimation in levels of Moderate to Vigorous Physical Activity (MVPA) due to the longer accelerometer epoch time which may not capture the sporadic PA commonly observed in children. Overall levels of PA were also low and this may have masked the effect of preterm birth; thus, it may be more optimal to study a younger cohort of children. We now report on objectively measured levels of physical activity and sedentary behavior from a UK-wide cohort of 7-year old preterm children born in the 2000’s who reflect the modern era of neonatal care including innovations such as the routine use of exogenous surfactant and antenatal corticosteroids. These interventions are likely to have improved the respiratory outcomes of preterm birth but during this time rates of CLD have stayed largely the same, perhaps due to increased survival at the extremes of gestation.

Other studies investigating PA and sedentary behavior in preterm-born children are further limited by the focus on low birthweight or children with chronic lung disease of prematurity (CLD, often called bronchopulmonary dysplasia, BPD), have used questionnaire-based data [11, 12], and are geographically based in relatively small areas. The aim of the study was to investigate if lower gestational age at birth is associated with reduced PA and increased sedentary behavior in 7-year old preterm-born children when compared to those born at term, and to investigate if this is mediated through an increase in respiratory symptoms.

## Methods

### Millennium Cohort Study

**Fig 1:**
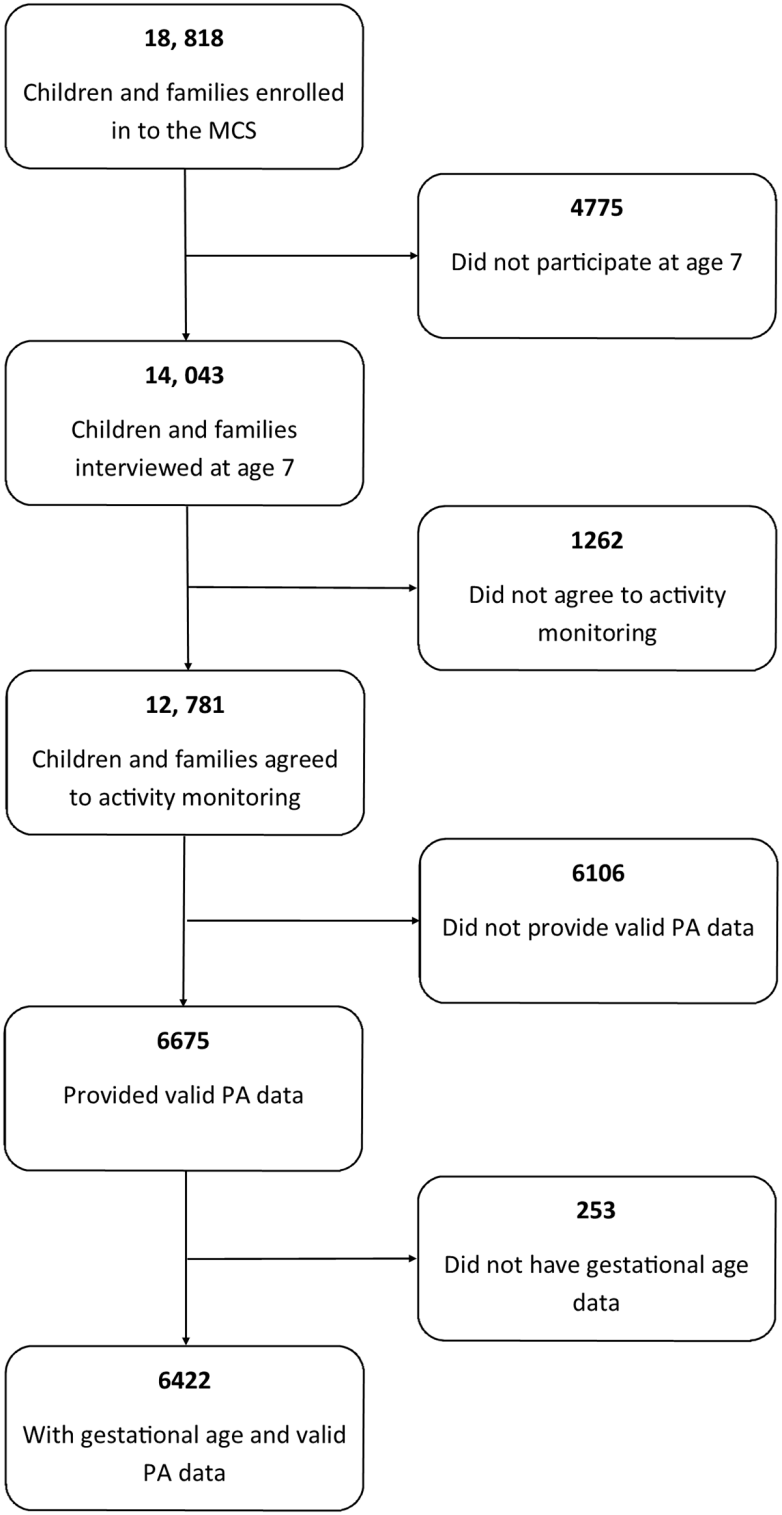
Flow diagram of MCS participants with available PA and gestational age data at age 7.

We used data from the Millennium Cohort Study (MCS), a UK-based geographically representative cohort which disproportionally sampled areas of ethnic minorities and lower socio-economic status in England as well as the devolved nations of the UK (Wales, Scotland and Northern Ireland) [13]. Briefly, 18,818 children and families were recruited between the years of 2000–2002 and followed up from the age of nine months and subsequently at 3, 5 and 7 years of age with data collection ongoing. At the age 7 follow-up, 14043 of 18818 families participated in the home interview where 12781 of 14043 participants (91%) agreed to wear the activity monitor [14]. Valid PA data (at least 10 hours on at least 2 days) were available for 6675 participants representing 52% of the children included at 7 years of age. After removing those with missing gestational age at birth (n = 253), 6422/12781 (50%) children were included (Fig 1). Written informed consent was obtained prior to data collection from the child’s caregiver. Ethics approval was given by the Northern and Yorkshire research ethics committee. The data are available as a downloadable resource and do not contain personal identifiable information.

### Physical Activity

Children were provided with a uniaxial accelerometer (Actigraph, Pensacola, Florida, USA) to wear around the waist during waking hours for 7 consecutive days. The device has been validated against criterion standards and the methods for data recording, processing and establishing activity ‘cut-point’ thresholds are described elsewhere [15]. Valid data were considered to be a recording of a minimum of 10 or more hours on 2 or more days of recording [15, 16].

We used three outcomes in our analysis: Total PA, the average number counts per minute (cpm) over the period of the valid recording; MVPA, defined as average time in minutes per day spent at >2241cpm over the period of the valid recording; and sedentary behavior, average time in minutes per day spent at <100cpm. Total PA was used as it provides a summary measure of activity and is the variable used for comparison in calibration studies [15]. MVPA and sedentary behavior were used because national guidelines recommend at least 60 minutes of MVPA per day and reductions in sedentary behavior [5].

### Perinatal data

Perinatal data on birthweight, admission to the neonatal intensive care unit and length of hospital stay were extracted from hospital records [17]. Gestational age was derived from maternal report of the expected due date during the 9 month interview, which was based on the date of the last menstrual period and results of the antenatal ultrasound scan. These data have been shown to provide an accurate estimation when compared to linked hospital database records [18]. Children with valid accelerometry data were divided into four groups based on gestational age at birth: 25–32, 33–34, 35–36 and 37–43 weeks’ gestation (term control). This approach is similar to our other studies on lung function [3] and physical activity [10] and that of others as it represents specific stages in lung development and children most at risk of lung disease [19, 20].

### Respiratory outcomes

Rates of wheeze and atopy in the last 12 months were derived from interviews conducted by the study team during the home visit at 7 years of age. Use of such data for assessing respiratory outcomes is well-established and extensively validated [21].

### Demographics

Information on gender, ethnicity, mother’s smoking habits during pregnancy and educational qualifications were collected at the home-based interview conducted 9 months after birth [22]. Highest level of education was categorized into none, other, GCSE (national school exams at 16), A-level (national school exams at age 18), or university degrees. At the age 7 home visit data on the child’s age, weight and height were collected, from which BMI was ascertained. Social class was based on the 5-category UK Office for National Statistics Socio-economic Classification (NS-SEC) which relates to maternal employment (last known job) at the age 7 visit.

### Statistical Analysis

Firstly, in order to assess the representativeness of the available data all demographic variables were compared between participants who provided valid PA data and those who did not, using χ^2^ tests.

Secondly, we compared birth characteristics in the four groups of gestation using ANOVA for normally distributed data and used Kruskal-Wallis Tests for non-normal data. Since total accelerometer wear time was not consistent across all participants, we used the methods of Griffiths to standardize the measures of MVPA and sedentary behavior. This was achieved by introducing a standard day of equal duration for all children (equal to the mean wear time across all valid days of recording) [23, 24]. Each PA variable was then tested for normality using P-P plots; MVPA was found to have a positively skewed distribution and was therefore square-root transformed prior to analysis. Total PA and sedentary behavior did not require transformation.

In order to investigate the effect of gestation group on measures of activity (Total, MVPA or sedentary behavior), we used a series of general linear models. Since our previous study showed a significant difference between levels of activity in males and females, we first tested the association between gestation group and PA for an interaction with gender. As expected there was significant evidence (p<0.001) of an interaction hence we have stratified all analyses by gender. In model 1 (adjusted for birth factors only) we included gestation group, single or multiple births, intrauterine growth restriction status (IUGR, defined as <10^th^ centile for standardised birthweight [LMS Grow Program; Medical Research Council, UK]), admission to the neonatal unit and maternal smoking in pregnancy; in model 2 we adjusted for the variables in model 1 plus age, body mass index (BMI) and season of accelerometry data collection at the 7-year visit; in model 3 we adjusted for the variables in model 2 plus social factors at the 7-year visit: ethnicity, maternal education and social class; in model 4 we adjusted for the variables in model 3 plus respiratory symptoms: current wheeze and atopy. General linear modelling was performed using SPSS Statistics (version 20, IBM, Chicago, IL., USA). In order to further investigate the mediation of respiratory symptoms (current wheeze and atopy) on the association between gestational age and measures of PA, we conducted mediation analysis. This enables distinction between the direct effect, which is the relationship of the independent variable on the dependent variable, adjusting for the mediating variable, and the indirect effect, which is the effect of the independent variable on the dependent variable through the mediating variable. Mediation is said to occur if the strength of the relationship between the predictor and the outcome is diminished by including a mediator [25]. Mediation analysis was performed with Mplus using structural equation modelling (version 7, Muthen and Muthen, Los Angeles, California USA). For all analyses p<0.05 was considered statistically significant.

## Results

After excluding participants without valid accelerometry and gestational age data, 79 children were born at 24–32 weeks’, 119 were born at 33–34 weeks’, and 275 were born at 35–36 weeks’ gestation. The control population consisted of 5949 children born at term (37–43 weeks’ gestation) (Table 1). Children born at ≤32 weeks’ gestational age were noted to have a significantly lower BMI and had increased incidence of recent wheeze and asthma when compared to those born at term. Rates of atopy were similar between gestation groups. Preterm born children were more likely to be born to mothers who smoked during pregnancy; however, there were no significant differences in socio-economic status when measured by maternal employment or maternal education.

**Table 1:** Characteristics of participants included who had valid PA data and gestational age data.

	25-32w	33-34w	35-36w	Term
Total Cohort	253	373	876	17063
Had PA data at age 7	79	119	275	5949
Gender, M (%)	37/79 (46.8)	57/119 (47.9)	135/275 (49.1)	2901/5949 (48.8)
Mean age, years (SD)	7.3 (0.3)	7.2 (0.2)	7.2 (0.2)	7.2 (0.2)
Mean Height, cm (SD)	122 (5.9)	122 (5.6)	123 (5.8)	124 (5.4)
Mean Weight, kg (SD)^§^	23.3 (4.4)	24.3 (4.2)	25.2 (5.1)	25.2 (4.5)
Mean BMI (SD) ^§^	15.5 (1.96)	16.2 (1.9)	16.5 (2.4)	16.4 (2.4)
Wheeze at 7 (%)^§^	17/79 (21.5)	12/119 (10.1)	34/274 (12.4)	661/5941 (11)
Asthma at 7 (%)^§^	19/79 (24.1)	24/118 (20.3)	47/272 (17.3)	878/5936 (14.8)
Atopy at 7 (%)	35/79 (44.3)	49/119 (41.2)	125/274 (45.6)	2446/5943 (41.4)
Neonatal characteristics	25-32w	33-34w	35-36w	Term
Mean Birthweight, g (SD) ^§^	1619 (614)	2147 (507)	2683 (503)	3452 (495)
IUGR^§^	9/79 (11.4)	22/119 (18.5)	25/274 (9.1)	522/5944 (8.8)
Caesarian section (%)^§^	44/79 (55.7)	60/119 (50.4)	102/275 (37.1)	1244/5922 (21.0)
Admitted to Neonatal unit (%)^§^	69 (87.3)	87 (73.1)	91 (33.1)	351 (5.9)
Length of stay after birth, days (SD) ^§^	44 (27.6)	19 (20.3)	8 (8.4)	3 (6.0)
Breastfeeding (%)^§^	58/79 (73.4)	81/119 (68.1)	187275 (68.0)	4454/5949 (74.9)
Socio-economic characteristics	25-32w	33-34w	35-36w	Term
Maternal smoking in pregnancy (%)^§^				
Smoking	24/78(30.8)	42/119 (35.3)	91/275(34.2)	1636/5944 (27.5)
Non-smoking	54/78 (69.2)	77/119 (64.7)	181/275 (65.8)	4308/5944 (72.5)
Employment status (%)				
Management/Professional	28/76 (36.8)	33/110 (30.0)	86/259 (33.2)	2119/5703 (37.2)
Intermediate	12/76 (15.8)	22/110 (20.0)	64/259 (24.7)	1115/5703 (19.6)
Self employed	7/76 (9.2)	11/110 (10.0)	15/259 (5.5)	446/5703 (7.8)
Supervisory/Technical	5/76 (6.6)	8/110 (7.3)	12/259 (4.6)	260/5703 (4.6)
Semi routine/routine	24/76 (31.6)	36/110 (32.7)	82/259 (31.7)	1763/5703 (30.9)
Ethnicity (%)				
White	68/79 (86.1)	105/119 (88.2)	247/275 (89.8)	5261/5938 (88.6)
Other	11/79 (13.9)	14/119 (11.8)	28/275 (10.2)	677/5938 (11.4)
Mother’s education (%)				
None of these	13/79 (16.5)	12/119 (10.1)	36/275 (13.1)	640/6418 (10.8)
Other academic	0 (0.0)	0/119 (0.0)	1/275 (0.4)	133/6418 (2.2)
GCSE grades D-G	6/79 (7.6)	15/119 (12.6)	23/275 (8.4)	520/6418 (8.7)
GCSE grades (A-C)	27/79 (34.2)	41/119 (34.5)	107/275 (38.9)	1916/6418 (32.2)
A or AS levels	5/79 (6.3)	10/119 (8.4)	22/275 (8.0)	676/6418 (11.4)
Diplomas in College	8/79 (10.1)	17/119 (14.3)	29/275 (10.5)	648/6418 (10.9)
First Degree	16/79 (20.3)	22/119 (18.5)	51/275 (18.5)	1118/6418 (18.8)
Higher degree	4/79 (5.1)	2/119 (2.2)	6/27 (2.2)	297/6418 (5.0)

^§^ p<0.05

There were some small but statistically significant differences between participants who provided valid PA data and those who did not. Those with valid data were more likely to be female, white, and born at term. Mothers were more likely to have achieved a higher educational qualification, have not smoked during pregnancy and be of higher socio-economic status (S1 table, online supplemental information). A previous study investigating the demography of non-consent to participate in the accelerometer study found consent was less likely for children with limiting illness or disability, those who reported exercise less than once per week, and a number of social factors related to increased levels of deprivation [26].

Table 2 shows levels of PA by gestation group, presented separately for males and females. Females participated in significantly less PA and spent more time in sedentary behaviour. The mean levels of MVPA were close to or exceeded the recommended guidelines of 60 minutes per day (Preterm females 55, males 66 min·day^-1^; Term females 55, males 69 min·day^-1^). 63% of term-born boys participated in greater or equal to 60 minutes of MVPA compared to 55% of those born preterm. In females, 34% of those born preterm participated in greater or equal to 60 minutes of MVPA compared to 36% of those born at term.

**Table 2:** Levels of physical activity by gestation groups adjusted for wear time.

	25-32w N = 79	33-34w N = 119	35-36w N = 275	All Preterm N = 473	Term N = 5949	Whole cohort N = 6422
Total PA (cpm)						
M	576 (145)	640 (175)	630 (161)	624 (143)	640 (154)	639 (155)
F	534 (159)	527 (163)	581 (152)	559 (157)	568 (142)	567 (144)
Sedentary (min·day^-1^)						
M	404 (45)	385 (48)	383 (50)	386 (49)	384 (49)	384 (49)
F	417 (51)	416 (52)	401 (53)	407 (53)	400 (53)	400 (50)
Light (min·day^-1^)						
M	271 (32)	281 (33)	285 (38)	282 (36)	282 (38)	282 (38)
F	265 (38)	269 (39)	276 (41)	407 (53)	400 (49)	279 (39)
MVPA (min·day^-1^)						
M	61 (23)	69 (25)	67 (24)	66 (24)	69 (23)	69 (23)
F	52 (21)	51 (22)	58 (20)	54 (21)	55 (19)	55 (19)

Data are means (SD)

The results of the main analyses, separated by gender, are shown in Table 3. In the minimally adjusted model (model 1), gestational age in boys was significantly associated with reduced levels of MVPA in the ≤32 week group when compared to term but not for the 33–34 and 35–36 week groups. After addition of further sets of variables, this relationship remained statistically significant in the fully adjusted model (model 4). Although the effect size of gestational age group and contribution to the model fit as a whole were both small, when we used the regression equation derived from model 4 to calculate the mean predicted values it equated to a difference of approximately 9 minutes per day of MVPA between boys born at term (mean predicted MVPA 67.7 min·day^-1^) and those born ≤32 weeks’ gestation (mean predicted MVPA 58.9 min·day^-1^)—over 1 hour in total of the course of a week. Total PA was also noted to be lower in the ≤32 week group when compared to term in model 1; however, these associations attenuated somewhat as further variables were added to the model. There was also a significant association between gestational age (≤ 32 weeks’ gestation) and increased levels of sedentary behavior for model 1 when compared to term-born controls; this again attenuated in models 2–4 mainly due to the strong influence of non-white ethnicity and higher socio-economic status, both of which were significantly associated with increased levels of sedentary behavior. Results from univariate analyses are available in S2 Table (supporting information). In females, there were no significant associations between gestation groups for Total PA, MVPA or sedentary behavior in the minimally or maximally adjusted models.

**Table 3:** Results of general linear models regressing activity variables on gestation groups adjusting for confounders.

MALES			Total PA			MVPA*			Sedentary		
		n	β	95% CI	p-value	β	95% CI	p-value	β	95% CI	p-value
Model 1	≤32	29	-67.2	-128, -5.91	0.03	-0.59	-1.13, -0.05	0.03	20.1	0.62, 39.5	0.04
	33–34	44	-1.92	-53.0, 49.1	0.94	-0.08	-0.53, 0.37	0.72	2.03	-14.2, 18.2	0.81
	35–36	104	-13.9	-46.0, 18.3	0.40	-0.19	-0.48, 0.09	0.19	0.73	-9.49, 11.0	0.89
	Term	2377	Ref			Ref			Ref		
Model 2	≤32	29	-61.6	-122, -1.37	0.05	-0.58	-1.12, -0.05	0.03	15.58	-3.57, 34.7	0.11
	33–34	44	-1.12	-51.1, 48.9	0.97	-0.09	-0.53, 0.36	0.70	1.22	-14.7, 17.13	0.88
	35–36	103	-13.2	-44.8, 18.5	0.41	-0.20	-0.48, 0.08	0.16	-0.31	-10.4, 9.77	0.95
	Term	2366	Ref			Ref			Ref		
Model 3	≤32	28	-59.8	-121, 1.58	0.06	-0.60	-1.15, -0.06	0.03	14.39	-5.01, 33.8	0.15
	33–34	43	7.06	-44.0, 58.1	0.79	-0.04	-0.49, 0.42	0.88	-0.25	-16.4, 15.9	0.98
	35–36	99	-13.7	-45.9, 18.5	0.41	-0.20	-0.49, 0.08	0.16	-0.23	-10.4, 10.0	0.96
	Term	2263				Ref			Ref		
Model 4	≤32	28	-58.7	-120, 2.81	0.06	-0.58	-1.12, -0.03	0.04	14.60	-4.85, 34.0	0.14
	33–34	43	6.93	-44.1, 58.0	0.79	-0.04	-0.49, 0.41	0.87	-0.36	-16.5, 15.8	0.97
	35–36	98	-13.9	-46.2, 18.5	0.40	-0.20	-0.49, 0.08	0.16	0.05	-10.2, 10.3	0.99
	Term	2261	Ref			Ref			Ref		
FEMALES	n		Total PA			MVPA*			Sedentary		
			β	95% CI	p-value	β	95% CI	p-value	β	95% CI	p-value
Model 1	≤32	27	-39.4	-98.5, 19.7	0.19	-0.27	-0.80, 0.24	0.30	18.01	-2.27, 38.3	0.08
	33–34	47	-26.0	-72.3, 20.4	0.27	-0.24	-0.65, 0.17	0.26	11.77	4.12, 27.6	0.15
	35–36	101	16.7	-13.3, 46.7	0.27	0.21	-0.05, 0.48	0.11	-2.18	-12.5, 8.10	0.68
	Term	2456	Ref			Ref			Ref		
Model 2	≤32	27	-43.2	-100, 14.0	0.14	-0.32	-0.83, 0.19	0.21	18.0	-1.47, 37.7	0.07
	33–34	47	-25.5	-70.3, 19.2	0.26	-0.24	-0.64, 0.16	0.24	11.2	-4.27, 26.6	0.16
	35–36	101	17.9	-11.1, 46.8	0.23	0.22	-0.04, 0.48	0.09	-2.62	-12.6, 7.37	0.61
	Term	2453	Ref			Ref			Ref		
Model 3	≤32	26	-35.6	-93.3, 22.1	0.23	-0.29	-0.81, 0.22	0.27	16.7	-3.16, 36.9	0.10
	33–34	40	0.83	-46.7,48.3	0.98	0.01	-0.42, 0.43	0.98	5.11	-11.3, 21.5	0.54
	35–36	92	7.73	-22.4,37.9	0.62	0.13	-0.14, 0.40	0.36	0.19	-10.2, 10.6	0.97
	Term	2323	Ref			Ref			Ref		
Model 4	≤32	26	-35.2	-92.9, 22.6	0.23	-0.29	-0.81, 0.23	0.27	16.9	-3.11, 36.9	0.10
	33–34	40	0.38	-47.2, 47.9	0.99	0.002	-0.43, 0.43	0.99	6.36	-10.1, 22.8	0.45
	35–36	92	8.00	-22.1, 38.1	0.60	0.13	-0.14, 0.40	0.36	0.40	-10.1, 10.9	0.94
	Term	2320	Ref			Ref			Ref		

Model 1: adjusted for single or multiple births, intrauterine growth restriction status, admission to the neonatal unit and maternal smoking in pregnancy

Model 2: as model 1 but additionally adjusted for body mass index, and season of accelerometry data collection

Model 3: as model 2 but additionally adjusted for ethnicity, maternal education and socio-economic status (mother’s last known employment)

Model 4: as model 3 but additionally adjusted for current wheeze, atopy

*MVPA beta values are square root transformed

**Fig 2:**
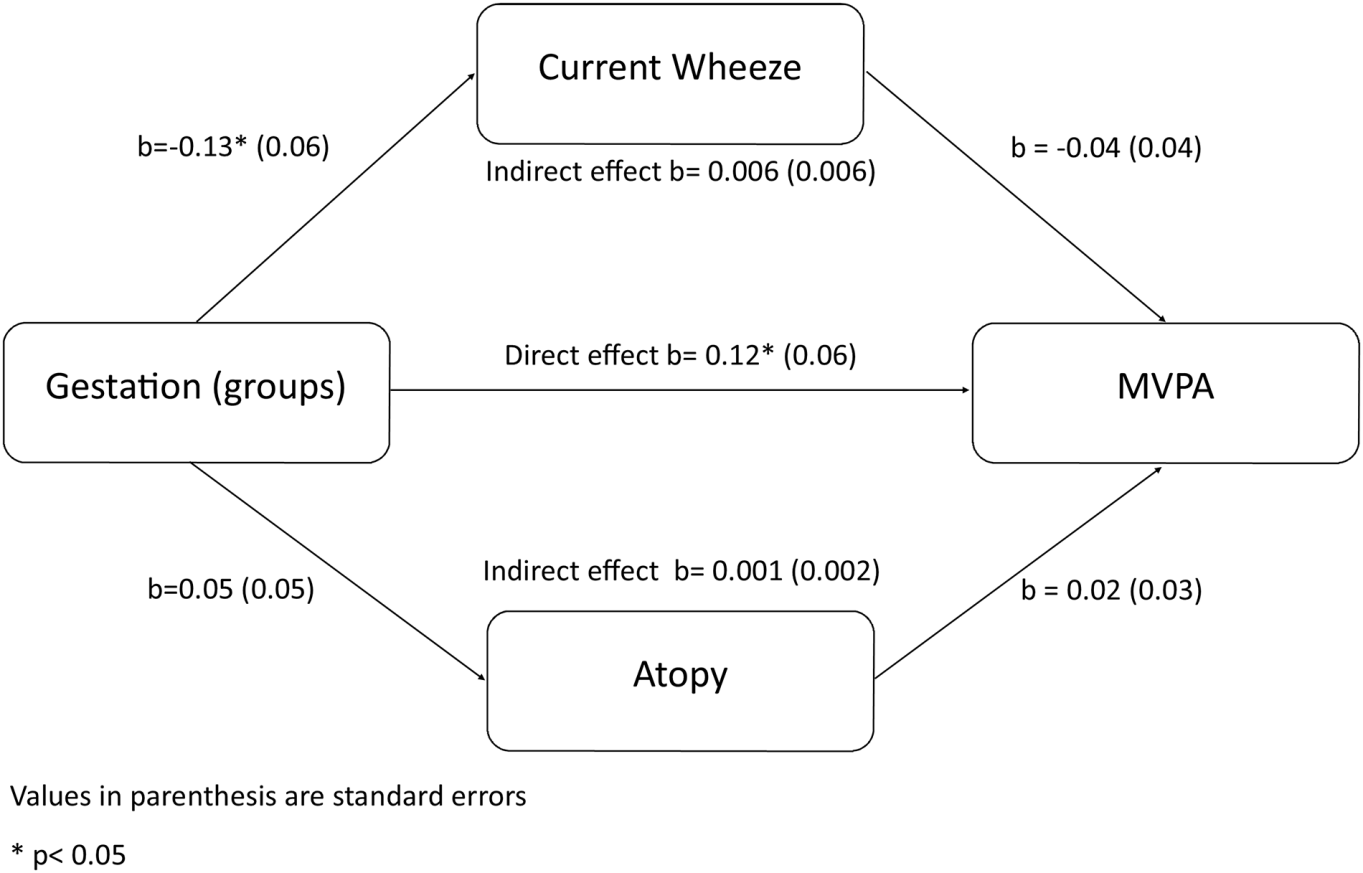
Mediation analysis of the effects of wheeze and atopy on the association between gestational age at birth and levels of MPVA in 7-year-old boys.

The results of the formal mediation analysis confirm the results as described above. Although gestational age was found to be a significant predictor of increased wheeze in boys, there was no evidence that wheeze or atopy moderates the association between gestational age and MVPA (indirect effect for wheeze β = 0.006 p = 0.3; indirect effect for atopy β = 0.001 p = 0.7: Fig 2). Figures representing mediation analyses for both males and females on both MVPA and sedentary behavior can be found online (S1–S3 Figs).

## Discussion

Using data from a large cohort study of children born during the surfactant era, we have shown that birth at ≤32 weeks’ gestation is a statistically significant factor in predicting reduced levels of MVPA in 7 year-old boys after adjustment for birth, growth and socio-economic factors. This equated to approximately a 1 hour difference in MVPA over the course of a week between boys born at ≤32 weeks’ gestation when compared to those born at term. There was also some association between decreased levels of total physical activity and increased levels of sedentary behaviour in boys born at ≤32 weeks’ gestation when compared to those born at term; these were not statistically significant in the fully adjusted models. None of the above findings were replicated in females.

We have recently shown no differences in Total PA, MVPA or sedentary behaviour in 11–15 year old preterm-born children when compared to term controls using data from the ALSPAC study. The decrements in lung function previously observed in preterm born children within this cohort were also not correlated with reduced activity. However, overall levels of activity were low [10]. In contrast, children participating in the current study showed significantly higher levels of MVPA, meeting or exceeding the recommended guidelines. In comparison, another UK-based cohort of 7 year old children conducted by Basterfield et al showed that only 10% of boys and 3% of girls met the national guidelines for MVPA when the cut-point was set at 3200cpm [27]. There are a number of possible reasons for these differences. Firstly, the 15 second data collection epoch used in the current study is more likely to detect MVPA, which occurs in short, sporadic bursts in young children [28]. This may be reflected in the differences in proportions of vigorous activity making up MVPA (32% of MVPA in MCS and 7% in ALSPAC). Secondly, the cut-point used to derive the MVPA variable are lower (>2241cpm) in the MCS, possibly leading to an over-estimation. When the thresholds used by Basterfield were applied to the MCS data, the proportion of boys and girls meeting MVPA guidelines fell to 14% and 0.4% respectively [23]. Longitudinal data from Basterfield et al also demonstrated a significant decrease in MVPA and a rise in sedentary behaviour between the ages of 7–9 years. One possible explanation for these differences is that younger children may be more likely to spend time in habitual ‘free-living’ play activities than 9 or 11 year olds who are undergoing a transition to middle/high school education with formalised physical education lessons and heighted social pressures.

Similarly, differences in sedentary behaviour between cohorts are difficult to compare due to variation in the accelerometer cut-point used, for example: <100cpm in the MCS, <200cpm in ALSPAC and <1100 in the Gateshead Millennium Study [27], the latter of which captures a proportion of light intensity PA as defined in the MCS or ALSPAC. Although we did show that the ≤32 week gestation group had increased levels of sedentary behaviour compared to term-born boys, this association was not robust to the addition of other explanatory variables in terms of maintaining statistical significance. However, this still does equate to over 14 minutes of additional sedentary time per day which could reflect a preference for this group of children to choose to spend less time active.

It is interesting that markers of lower socio-economic status (maternal smoking history) were associated with less time spent in sedentary behaviour and increases in total PA and MVPA. In contrast, markers of higher status (mother’s last known employment, higher level of maternal education) where associated with decreased Total PA, decreased MVPA, and increased sedentary time. Non-white ethnicity was also significantly associated with lower Total PA and more time spent sedentary. The observed differences between socio-economic groups were small and without a clear trend, consistent with previous studies [29]. More research is warranted in this area to determine the reasons for differences in PA between socio-economic and ethnic groups.

In our recent study, we observed increased rates of exercise-induced bronchoconstriction and reduced levels of PA in preterm-born children using data from a self-completed questionnaire [30]. This lead us to the hypothesis proposed in this study that experiencing such symptoms of airway obstruction on exertion is a conceivable pathway to reduced levels of activity. A previous study described that children with asthma may exercise less to avoid the unpleasantness of these sensations [31]. Other questionnaire-based studies also support the hypothesis that preterm-born children consider themselves less active. Clemm et al. and Welsh et al. demonstrated both reduced levels of self-reported PA, however both these studies included only children with gestation ≤28 weeks' gestation [32, 33]. Furthermore, in the latter study, questionnaire responses did not correlate with accelerometry which may be attributed to the differing types of activity recorded when using subjective or objective methods [28].

Somewhat surprisingly, our findings suggest that despite preterm-born children demonstrating significantly increased rates of current wheeze, this was not a mediating factor in the relationship between gestational age and levels of physical activity. It is likely that the reporting of respiratory symptoms at the time of assessment was accurate as the questionnaire used in the MCS is well validated and collected during a home visit where answers could be clarified. Although it is difficult to compare our study with those focusing on low birthweight, such studies often contain a significant proportion of participants who were born preterm, especially in the very low birthweight and extremely low birthweight groups (<1500g and <1000g birthweight, respectively). A summary of these studies showed that reductions in exercise capacity were present from an early age—consistent with the modest differences found in our recent systematic review [4]. This may reflect the ability of the cardio-respiratory system to compensate for any airway obstruction during exertion, especially due to the increased ventilatory reserve available in children [30]; however this is accompanied with a diminished ability to perform tests of motor function and strength-based tasks, with differences in habitual physical activity only manifesting from adolescence onwards [34]. Other potential mediators of PA in preterm-born children therefore may include differences in body composition such as muscle mass and fat mass, and limitations imposed by neurodevelopmental sequelae of preterm birth [35, 36]. Similar reasons could possibly help to explain why in this study, we found lower levels of MVPA in preterm-born boys ≤32 weeks’ gestation but not in girls. Peacock *et al* suggested that respiratory and neurological outcomes following preterm birth may be worse in males than in females, especially at the extremes of gestation [37]. Also, sex-typed behaviour emerges early in childhood and ‘male typical’ activities have been linked to increased levels PA by late childhood [38]; it is conceivable that preterm-born boys with milder forms of developmental coordination disorder, which has been linked to decreased MVPA, spend less time in male typical activities due to reduced self-perception of abilities and difficulties with keeping up with their peers [39].

Notwithstanding, since PA has the potential to improve cardio-respiratory health, preterm-born children should be encouraged to participate in adequate levels in order to develop positive attitudes towards exercise. Although the 9-minute difference per day in boys of ≤32 weeks’ gestation was small, this equated to over 1 hour a week and this may have some clinical utility. An increase of 9 minutes would improve the proportion of preterm-born boys meeting daily MVPA recommendations from 55% to approximately 73%. Achieving such improvements may be of heightened importance given the clear evidence of a steep decline in PA which has been observed during adolescence and consistent reporting of decrements in exercise capacity and PA in preterm-born adults. Both point towards detraining of the cardio-respiratory system which may have implications for the development of early-onset chronic respiratory disease and thus deserves further surveillance.

The main strengths of our study are the use of recent data from a large contemporary cohort which sampled from the whole of the UK and from a diverse range of socio-economic backgrounds. The Actigraph device used has been well validated and in contrast to other studies used a shorter sampling epoch thus allowing for capture of shorter periods of MVPA. We acknowledge that a limitation is the accelerometer required removing for contact sports or water-based activities, and does not capture bicycle riding adequately. However, the age of children in this study makes participation in contact sports less likely than those conducted in older cohorts. In any case, our data reflect habitual activity rather than sports participation. Additionally, our sample was taken from a proportion of the MCS who participated in the home visit and provided valid accelerometry data; the study by Rich et al showed that non consent rates were higher in children with a limiting illness or disability [26]. Such selection bias reduces the possibility of including children with severe physical or cognitive impairments. In common with many longitudinal studies, there is some attrition over time and we are limited by the data available. Other data on socio-economic status such as the Index of Multiple Deprivation are available but this is calculated differently in the four devolved nations of the UK. Although our participants were of higher socio-economic status we believe our results are generalizable as lower socio-economic status was associated with increases in physical activity [23]. Finally, although banding of gestation in to categories will result in a small degree of misclassification, modelling measures of PA on a continuous measure of gestational age (in completed weeks) gave a poor linear fit. This was only marginally improved by fitting splines to the gestational age data similar to an approach we recently used [Watkins WJ, *in press*). Since our previous work also demonstrates non-linear decrements in lung function between groups and also reflects distinct stages of lung development we feel such an approach is valid.

In conclusion, we have shown small but important differences in objectively measured physical activity in 7-year old boys who were born very preterm when compared to term-born controls. Children born in the MCS reflect the more immature graduates of modern neonatal care including gentler ventilation, antenatal steroids and exogenous surfactant therapy. The difference in cut-points for levels of activity make comparisons of similar studies difficult; however, it appears that preterm-born children are capable of participating in adequate levels of habitual physical activity. This requires surveillance due to the declines in PA and increased sedentary behaviour that have been shown to occur throughout childhood and adolescence.

## Supporting Information

S1 Fig Mediation analysis of the effects of wheeze and atopy on the association between gestational age at birth and levels of MPVA in 7-year-old girls. (TIF)

S2 Fig Mediation analysis of the effects of wheeze and atopy on the association between gestational age at birth and levels of sedentary behavior in 7-year-old boys. (TIF)

S3 Fig Mediation analysis of the effects of wheeze and atopy on the association between gestational age at birth and levels of sedentary behavior in 7-year-old girls. (TIF)

S1 Table Demographics of children participating in accelerometry. (DOCX)

S2 Table Univariate analysis of confounders and their association with levels of physical activity. (DOCX)

## Abbreviations

ALSPAC: Avon Longitudinal Study of Parents and Children
BMI: Body mass index
CLD: Chronic lung disease of prematurity
Cpm: Counts per minute
MCS: Millennium Cohort Study
MVPA: Moderate to vigorous physical activity
PA: Physical activity
