# Supplementary figures and images for: Physical Activity and Sedentary Behavior in Preterm-Born 7-Year Old Children
Source: PLoS One. 2016 May 11;11(5):e0155229. doi: 10.1371/journal.pone.0155229 (PMC4864195; doi:10.1371/journal.pone.0155229)

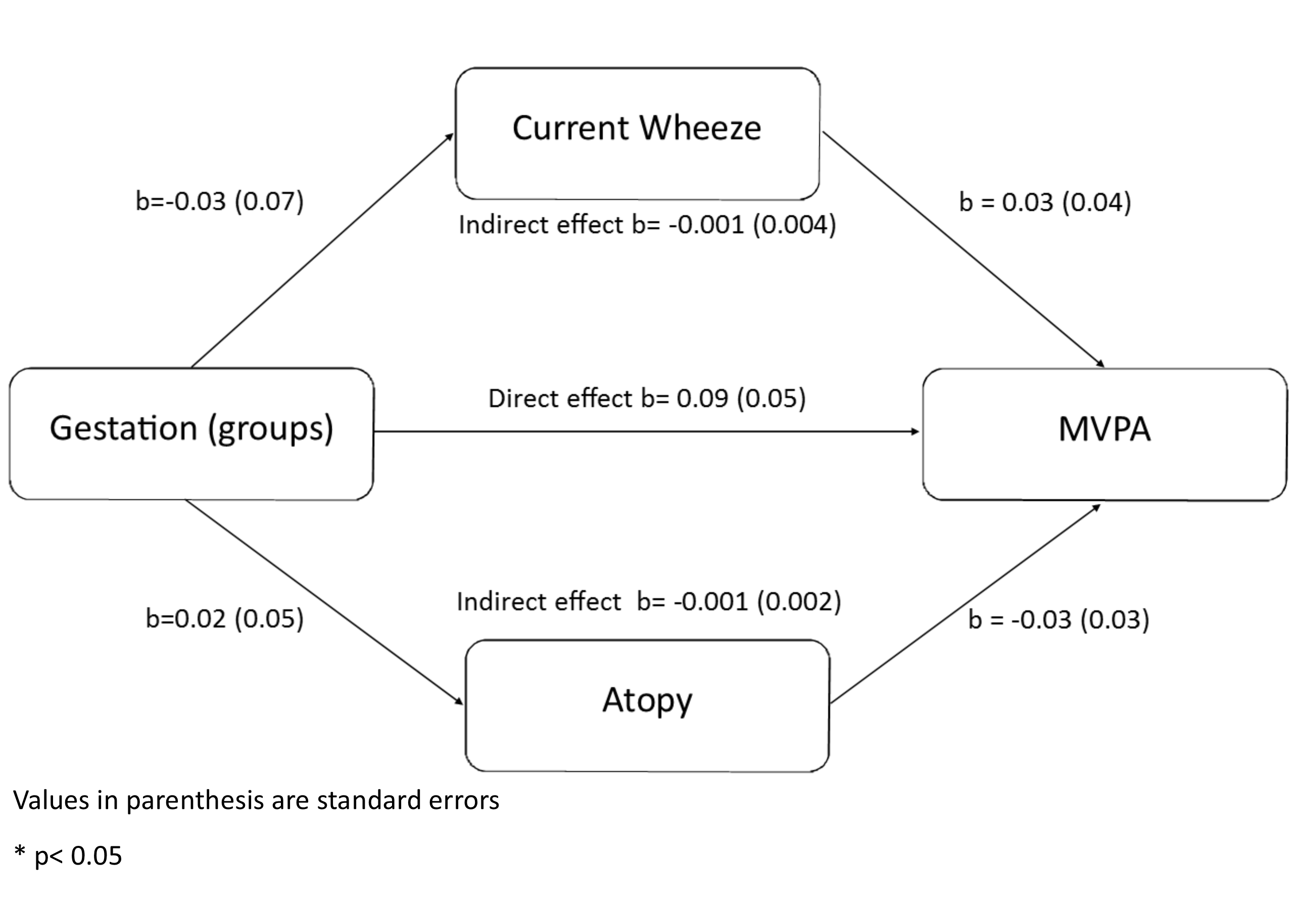

Supplement: S1 Fig — (TIF) [file pone.0155229.s001.tif]

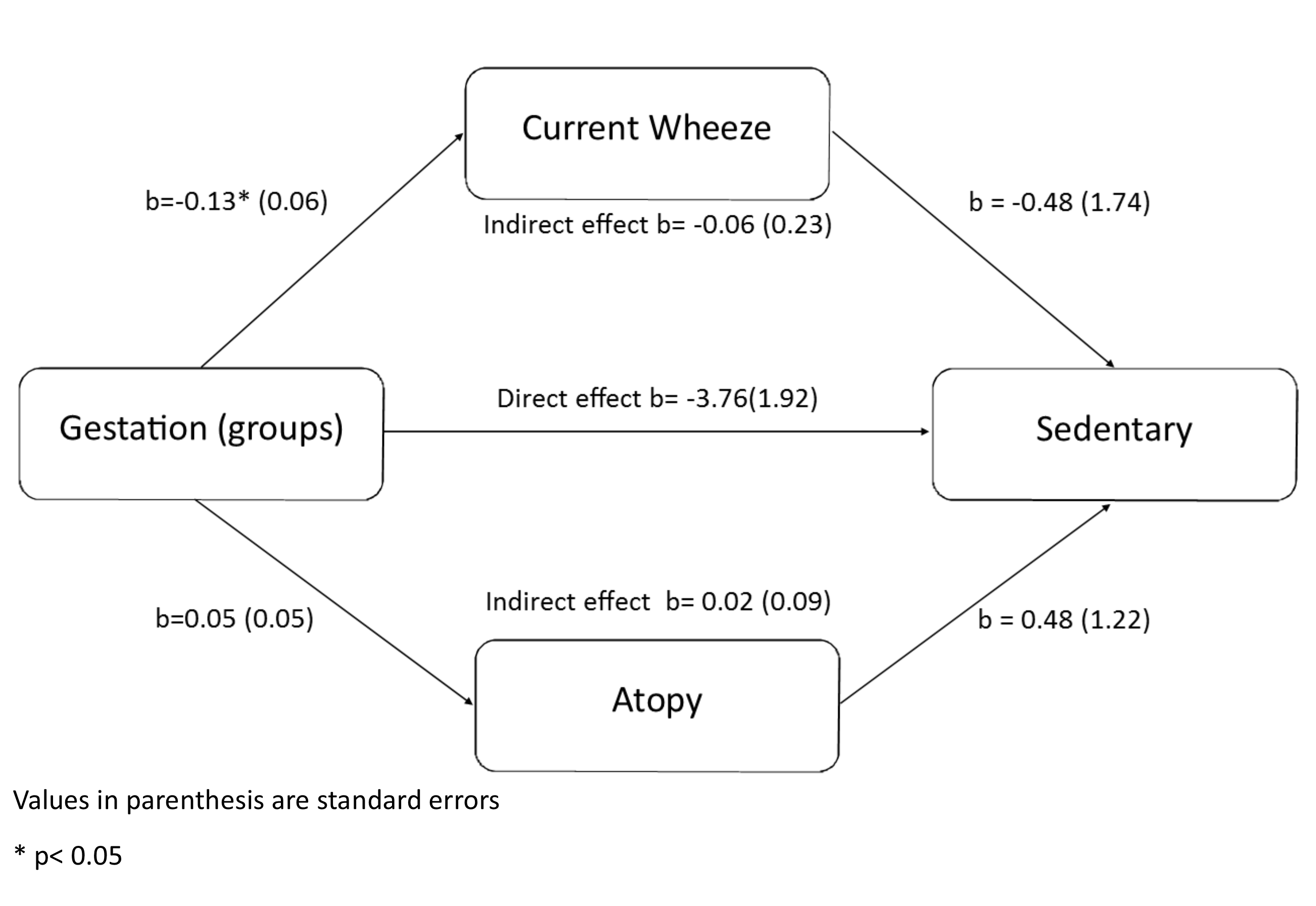

Supplement: S2 Fig — (TIF) [file pone.0155229.s002.tif]

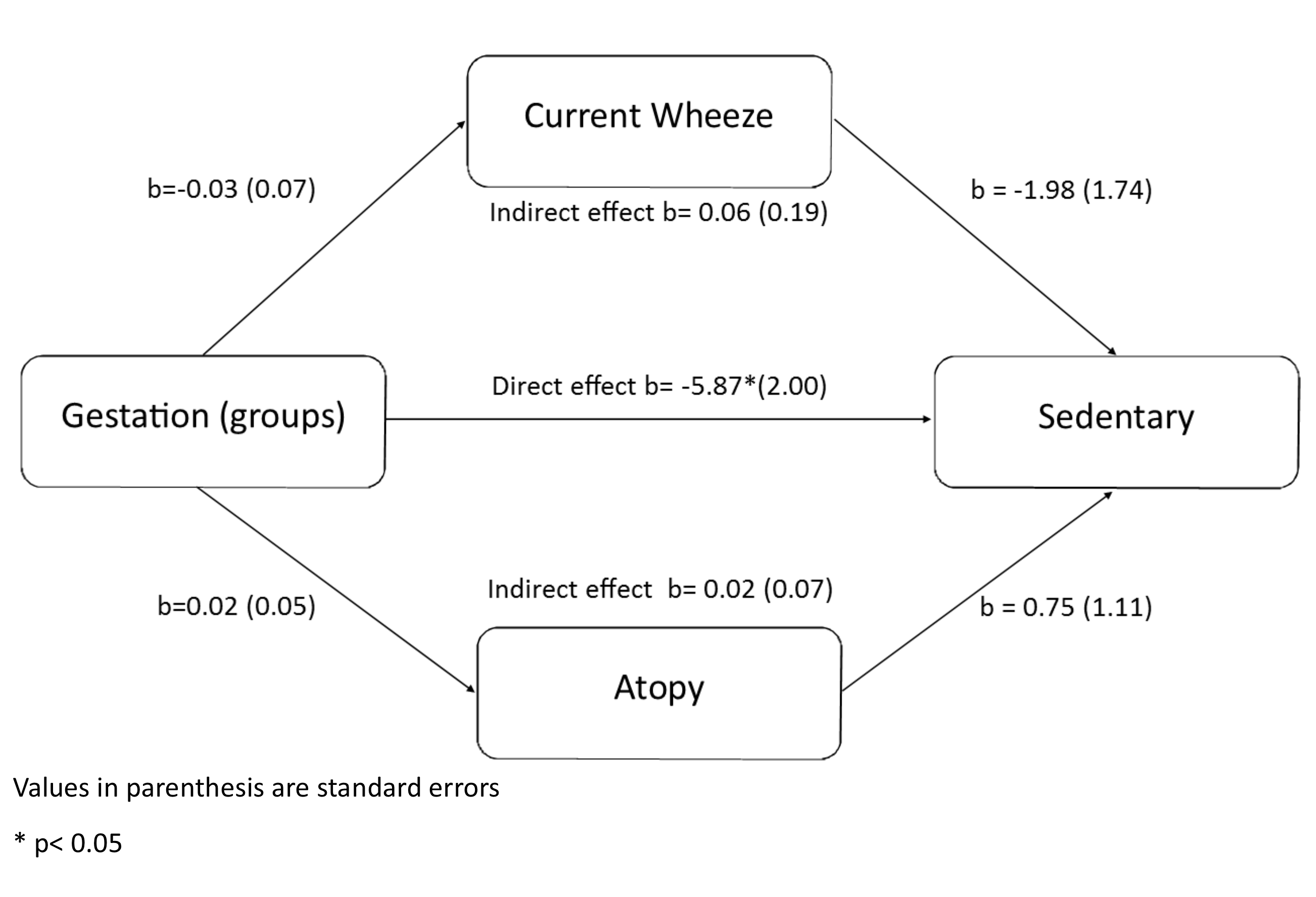

Supplement: S3 Fig — (TIF) [file pone.0155229.s003.tif]
