# Supplementary material for: Physical Activity and Sedentary Behavior in Preterm-Born 7-Year Old Children
Source: PLoS One. 2016 May 11;11(5):e0155229. doi: 10.1371/journal.pone.0155229 (PMC4864195; doi:10.1371/journal.pone.0155229)
Supplement: S1 Table — (DOCX) [file pone.0155229.s004.docx]

|  | Participants  N= 6422 | Non-participants  N= 12842 |
| --- | --- | --- |
| Sex (%)* |  |  |
| Male | 3130/6422 (48.7) | 6489/12336 (52.6) |
| Female | 3292/6422 (51.3) | 5847/12336 (47.4) |
| Maternal smoking in pregnancy (%)^§^ |  |  |
| Smoking | 1796/6416 (28.0) | 4726/12294 (38.4) |
| Non-smoking | 4662/6416 (72.0) | 7568/12294 (61.6) |
| Employment status (%)^§^ |  |  |
| Management/Professional | 2337/6012 (38.9) | 2565/10546 (24.3) |
| Intermediate | 1172/6012 (19.5) | 1965/10546 (18.6) |
| Self employed | 274/6012 (4.6) | 384/10546 (3.6) |
| Supervisory/Technical | 352/6012 (5.9) | 654/10546 (6.2) |
| Semi routine/routine | 1877/6012 (31.2) | 4978/10546 (47.2) |
| Ethnicity (%)^§^ |  |  |
| White | 5681/6411 (88.6) | 9800/12300 (79.7) |
| Other | 730/6411 (11.4) | 2500/12300 (20.3) |
| Mother’s education (%)^§^ |  |  |
| None of these | 701/6418 (10.9) | 2941/12272 (24.0) |
| Other academic | 134/6418 (2.1) | 399/12272 (3.3) |
| GCSE grades D-G | 564/6418 (8.8) | 1424/12272 (11.6) |
| GCSE grades (A-C) | 2091/6418 (32.6) | 4166/12272 (33.9) |
| A or AS levels | 713/6418 (11.1) | 1024/12272 (8.3) |
| Diplomas in College | 702/6418 (10.9) | 885/12272 (7.2) |
| First Degree | 1204/6418 (18.8) | 1117/12272 (9.1) |
| Higher degree | 309/6418 (4.8) | 316/12272 (2.6) |
| Gestational age (%)* |  |  |
| Term | 5949 (92.6) | 11114/12143 (91.5) |
| 35-36 | 275 (4.3) | 601/12143 (5.0) |
| 33-34 | 119 (1.9) | 245/12143 (2.0) |
| 25-32 | 79 (1.2) | 183/12143 (1.5) |
| * χ^2^< 0.05; ^§^ χ^2^= <0.001 | | |
