# Supplementary material for: Physical Activity and Sedentary Behavior in Preterm-Born 7-Year Old Children
Source: PLoS One. 2016 May 11;11(5):e0155229. doi: 10.1371/journal.pone.0155229 (PMC4864195; doi:10.1371/journal.pone.0155229)
Supplement: S2 Table — (DOCX) [file pone.0155229.s005.docx]

|  | Total PA |  |  | MVPA |  |  | Sedentary |  |  |
| --- | --- | --- | --- | --- | --- | --- | --- | --- | --- |
|  | B | 95% CI | p-value | B | 95% CI | p-value | B | 95% CI | p-value |
| SDS Birthweight | -0.67 | -4.28, 2.94 | 0.72 | -0.01 | 0.05, 0.02 | 0.45 | -0.95 | -2.13, 0.23 | 0.11 |
| IUGR |  |  |  |  |  |  |  |  |  |
| <10^th^ Centile | -4.15 | -17.56, 9.26 | 0.54 | -0.05 | -0.17, 0.07 | 0.43 | 0.72 | -3.64, 5.08 | 0.75 |
| ≥10^th^ Centile | Ref |  |  | Ref |  |  | Ref |  |  |
| Singleton |  |  |  |  |  |  |  |  |  |
| Singleton | 14.67 | -8.50, 37.84 | 0.22 | 0.99 | -0.12, 0.31 | 0.37 | -6.01 | -13.8, 1.73 | 0.13 |
| Multiple | Ref |  |  |  |  |  | Ref |  |  |
| Gender |  |  |  |  |  |  |  |  |  |
| Male | 72.04 | 64.77, 79.31 | <0.001 | 0.88 | 0.82, 0.95 | <0.001 | -16.5 | -19.0, -14.11 | <0.001 |
| Female | Ref |  |  | Ref |  |  | Ref |  |  |
| NNU Admission |  |  |  |  |  |  |  |  |  |
| Yes | -12.14 | -25.02, 0.74 | 0.07 | -0.11 | -0.22, 0.01 | 0.07 | 4.40 | 0.17, 8.62 | 0.04 |
| No | Ref |  |  | Ref |  |  | Ref |  |  |
| Mat Smoking |  |  |  |  |  |  |  |  |  |
| Yes | 18.88 | 10.56, 27.21 | <0.001 | 0.13 | 0.06, 0.21 | 0.001 | -3.50 | -6.23, 0.76 | 0.01 |
| No | Ref |  |  | Ref |  |  | Ref |  |  |
| Social Class (Mothers last employment) |  |  |  |  |  |  |  |  |  |
| Manage & Prof | -19.92 | -29.19, -10.66 | <0.001 | -0.125 | -0.21, -0.04 | 0.004 | 6.74 | 3.70, 9.78 | <0.001 |
| Intermediate | -11.37 | -22.50, -0.25 | 0.05 | -0.08 | -0.18, 0.02 | 0.10 | 3.68 | 0.10, 7.27 | 0.04 |
| Small emp/self em | -33.98 | -53.31, -16.65 | 0.001 | -0.16 | -0.30, -0.02 | 0.03 | 2.29 | -2.70, 7.29 | 0.40 |
| Lo sup/technical | -19.10 | -36.46, -1.75 | 0.03 | -0.02 | -0.19, 0.16 | 0.86 | 1.59 | -4.61, 7.80 | 0.62 |
| Routine | Ref |  |  | Ref |  |  | Ref |  |  |
| Ethnicity |  |  |  |  |  |  |  |  |  |
| White | 30.32 | 18.67, 41.98 | <0.001 | 0.07 | -0.03, 0.18 | 0.18 | -4.63 | -8.49, -0.76 | 0.02 |
| Other | Ref |  |  | Ref |  |  | Ref |  |  |
| Age | -19.98 | -34.94, -5.03 | 0.009 | 0.08 | -0.22, 0.06 | 0.27 | 11.93 | 6.96, 16.9 | <0.001 |
| Wheeze at 7 |  |  |  |  |  |  |  |  |  |
| Yes | 8.25 | 3.35, 19.84 | 0.16 | 0.06 | -0.05, 0.17 | 0.30 | -3.46 | -7.34, 0.42 | 0.08 |
| No | Ref |  |  | Ref |  |  | Ref |  |  |
| BMI at 7 | -4.51 | -6.24, 2.79 | <0.001 | -0.06 | -0.08, -0.04 | <0.001 | -0.35 | -0.93, 0.23 | 0.24 |
| Season of accelerometry |  |  |  |  |  |  |  |  |  |
| Winter | -100 | -115, -85.4 | <0.001 | -0.81 | -0.95, -0.68 | <0.001 | 28.1 | 23.2, 32.9 | <0.001 |
| Autumn | -89.6 | -102, -76.8 | <0.001 | -0.68 | -0.79, -0.56 | <0.001 | 27.4 | 23.2, 31.6 | <0.001 |
| Summer | -34.0 | -46.23, -21.6 | <0.001 | -0.32 | -0.43, -0.21 | <0.001 | 6.99 | 2.96, 11.0 | 0.001 |
| Spring | Ref |  |  | Ref |  |  | Ref |  |  |
| Mothers Education |  |  |  |  |  |  |  |  |  |
| None | 26.44 | 5.95, 46.94 | 0.01 | 0.17 | -0.02, 0.35 | 0.08 | -17.0 | -23.7, -10.3 | <0.001 |
| Other | -28.35 | -59.36, 2.70 | 0.07 | -0.25 | -0.53, 0.04 | 0.09 | 3.42 | -6.7, 13.6 | 0.51 |
| GCSE G-D | 22.07 | 0.83, 43.31 | 0.04 | 0.09 | -0.10, 0.28 | 0.35 | -11.5 | -18.5, -4.61 | 0.001 |
| GCSE A-C | 13.66 | -4.64, 31.95 | 0.14 | 0.01 | -0.16, 0.17 | 0.94 | -11.0 | -16.5, -5.00 | <0.001 |
| A/AS level | -2.16 | -22.60, 18.28 | 0.84 | -0.11 | -0.30, 0.08 | 0.25 | -4.32 | -11.0, 2.36 | 0.21 |
| Higher diploma | 5.82 | -14.67, 26.31 | 0.58 | -0.05 | -0.23, , 0.14 | 0.61 | -7.17 | -13.9, -0.48 | 0.04 |
| First Degree | -0.87 | -20.01, 18.27 | 0.93 | -0.06 | -0.24, 0.11 | 0.48 | -4.52 | -10.8, 1.73 | 0.16 |
| Higher degree | Ref |  |  | Ref |  |  | Ref |  |  |
